# Peripheral immune cells and glycation indices as potential diagnostic biomarkers in amyotrophic lateral sclerosis

**DOI:** 10.3389/ebm.2026.10987

**Published:** 2026-05-13

**Authors:** Xue Yang, Jing Yang, Rui Li, Hui Dong, Yaling Liu

**Affiliations:** 1 Department of Neurology, Hebei Medical University, Shijiazhuang, Hebei, China; 2 Department of Neurology, The Second Hospital of Hebei Medical University, Shijiazhuang, China; 3 Department of Neurology, Affiliated Hospital of Hebei Engineering University, Handan, China; 4 Key Laboratory of Clinical Neurology, Ministry of Education, Hebei Medical University, Shijiazhuang, China; 5 Neurological Laboratory of Hebei Province, Shijiazhuang, China

**Keywords:** amyotrophic lateral sclerosis, biomarkers, glycated haemoglobin A1c, glycosylation index, leukocyte

## Abstract

The diagnosis of amyotrophic lateral sclerosis (ALS) mainly relies on clinical symptoms and the exclusion of other diseases, with a lack of specific biomarkers, leading to delayed diagnosis and a high rate of misdiagnosis. This study aims to explore the utility of peripheral immune cells and glycosylation indices as potential diagnostic biomarkers for ALS to enhance the accuracy and efficiency of early ALS diagnosis. This retrospective study included 54 ALS patients diagnosed in our hospital from June 2023 to October 2024, along with 54 healthy controls. Blood samples and laboratory data, including levels of peripheral immune cells and glycosylation indices, were collected from both groups. Through logistic regression, random forest models, receiver operating characteristic (ROC) curve analysis, and SHAP interpretability analysis, the predictive abilities and clinical significance of each candidate indicator were screened and evaluated. Notable disparities were detected in age, leukocyte count, monocyte levels, glycated haemoglobin A1c (HbA1c), and haemoglobin glycation index (HGI) between the control and ALS groups (all *P* < 0.05). Logistic regression analysis revealed that age (OR = 1.114) and monocyte (OR = 3.174) were risk factors for ALS, while leukocyte (OR = 0.533) and HbA1c (OR = 0.069) were protective factors. The random forest algorithm, ranked by decreasing importance, showed that leukocyte, HGI, monocyte, and HbA1c level all influenced ALS. Using these indicators to predict ALS resulted in a false-positive rate of 18% and a false-negative rate of 6%. ROC curve analysis indicated that the combined use of leukocyte, monocyte, HbA1c level, and HGI provided the highest diagnostic value for ALS (AUC = 0.774), which was higher than that of any individual indicator (all *P* < 0.05). SHAP analysis visualization demonstrated that increased monocyte and decreased leukocyte, HGI, and HbA1c level were all associated with an increased risk of ALS onset, ranked in descending order of feature importance as monocyte, leukocyte, HGI, and HbA1c. Peripheral blood white blood cells, monocytes, HbA1c, and HGI can serve as potential diagnostic biomarkers for ALS. Combined detection can improve the diagnostic accuracy of ALS, facilitating early diagnosis and intervention, and ultimately improving patient prognosis. Further validation in cohorts including disease controls is required to confirm specificity.

## Impact statement

The diagnosis of ALS is notoriously delayed due to the lack of specific biomarkers, often relying on clinical exclusion. This study demonstrates that readily available peripheral blood markers (leukocyte and monocyte counts, HbA1c, and HGI) hold significant diagnostic value. The combined use of these indicators, reflecting both inflammatory and metabolic pathways implicated in ALS, provides a practical and minimally invasive approach to improve diagnostic accuracy. This biomarker panel could facilitate earlier identification of ALS in clinical practice, enabling timely intervention and potentially improving patient management and prognosis.

## Introduction

Amyotrophic lateral sclerosis (ALS) is a progressive neurodegenerative disease characterized by the degeneration of both upper and lower motor neurons in the brain and spinal cord [1]. Current estimates indicate an annual incidence rate of approximately 2 per 100,000 people and a prevalence of 6–9 per 100,000 [2]. In China, the annual incidence and prevalence rates are approximately (1.33–2.01) per 100,000 people and (2.31–3.58) per 100,000 people, respectively [3]. ALS typically strikes between the ages of 60 and 79 and is invariably fatal [4]. ALS is characterized by progressive muscle weakness and atrophy of the trunk, limbs, pharyngeal muscles, and respiratory muscles, with high disability and mortality rates [5]. Despite continuous in-depth research on ALS in recent years, its exact etiology and pathogenesis remain incompletely understood, and there is currently a lack of effective treatment methods [6]. The diagnosis of ALS primarily relies on clinical manifestations, electrophysiological examinations (such as electromyography), and imaging examinations. However, these methods have certain limitations [7]. The non-specificity of clinical manifestations and their similarity to other neuromuscular diseases make the diagnosis of ALS often challenging, resulting in high rates of diagnostic delay and misdiagnosis [8]. Although electrophysiological and imaging examinations are helpful for diagnosis, they do not provide specific biomarkers for the early identification of ALS [9]. Therefore, the search for biomarkers with high specificity and sensitivity is of great significance for the early diagnosis and disease monitoring of ALS.

The pathogenesis of ALS remains unclear, with neuroinflammation being recognized as a significant contributor to disease progression, in which the peripheral immune system plays a crucial role [10]. Subsets of innate immune cells, including monocytes/macrophages, natural killer cells, mast cells, dendritic cells, and neutrophils, are all implicated in the pathogenesis of ALS [11]. Previous studies have demonstrated that ALS patients with a higher neutrophil-to-lymphocyte ratio have a shorter survival time, and monocytes are associated with inflammatory responses [12]. Clinical indicators of glucose metabolism are significantly correlated with ALS progression. Research shows that ALS patients exhibit altered serum IgG glycosylation, characterized by high sialylation and low core fucosylation. A specific glycan, A2BG2, enhances antibody-dependent cellular cytotoxicity by increasing IgG’s affinity for CD16 on effector cells, suggesting its potential role as a biomarker in neuronal damage [13]. Furthermore, a prospective cohort study suggested that a diet higher in glycemic index and load may slow ALS progression [14]. Therefore, peripheral immune cell counts and glycosylation indices may serve as potential diagnostic biomarkers for ALS.

It is noteworthy that when analyzing the combined diagnostic value of multiple indicators, traditional statistical methods are often constrained by the assumption of linear relationships, making it difficult to capture the complex interactions among indicators. Moreover, they have stringent requirements for data distribution and are prone to model misspecification bias when handling non-linear, high-dimensional data commonly encountered in clinical settings. In contrast, machine learning prediction models demonstrate greater flexibility and adaptability. Random forest, as an ensemble learning method, effectively captures non-linear associations and interaction effects among variables by constructing multiple decision trees and integrating their predictive results. It does not require pre-specified data distribution forms, making it better suited to the complex characteristics of clinical data [15]. Additionally, through Bootstrap resampling and random feature selection, random forest reduces the risk of overfitting to a certain extent and enhances model stability in scenarios with limited sample sizes. However, its generalization ability still needs to be evaluated through cross-validation and external validation [16]. Further integration with SHAP interpretability analysis enables the quantification of the contribution of each candidate indicator to the prediction results at the model level and visually displays the direction and strength of its association with the risk of ALS onset. This not only facilitates the screening of potential key biomarkers but also enhances the transparency of the model’s predictive logic, making it easier for clinical researchers to understand and validate. It provides support for the clinical translation and application of the model.

This study aims to explore the potential of peripheral immune cells and glycosylation indices as potential diagnostic biomarkers for ALS through retrospective analysis. To screen valuable biomarkers for ALS diagnosis, this study included 54 confirmed ALS patients and 54 healthy controls. It analyzed peripheral immune cell counts and glycosylation index levels and comprehensively evaluated the predictive ability and clinical significance of each indicator by integrating logistic regression, random forest models, receiver operating characteristic (ROC) curves, and SHAP interpretability analysis. The objective of this study is to provide new ideas and methods for the early diagnosis of ALS, improve the diagnostic accuracy and early identification ability of ALS, thereby improving patient prognosis.

## Materials and methods

### Study subjects

**FIGURE 1:**
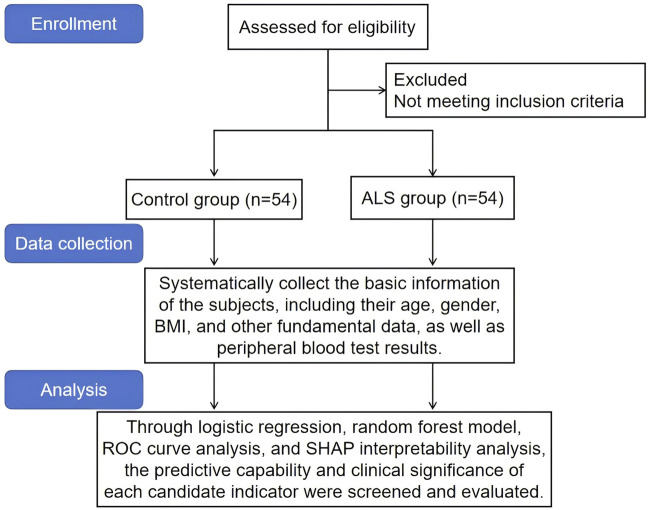
Research flowchart.

This study was a retrospective clinical controlled study. A total of 54 ALS patients diagnosed in our hospital from June 2023 to October 2024, along with 54 healthy controls, were included. Blood samples and laboratory data, including peripheral immune cell counts and glycosylation index levels, were collected from subjects in both groups. To screen valuable biomarkers for ALS diagnosis, this study comprehensively evaluated the predictive ability and clinical significance of each candidate indicator by integrating logistic regression, random forest models, ROC curves, and SHAP interpretability analysis. The detailed research process is shown in Figure 1.

### Inclusion criteria

ALS Group: (1) All patients were diagnosed according to the revised El Escorial criteria (Airlie House criteria), which remain the gold standard for ALS diagnosis [17]. Diagnosis was confirmed through a combination of clinical neurological examination, electromyography (EMG), neuroimaging (MRI of the cervical spine and brain), and comprehensive laboratory tests to exclude ALS-mimicking conditions. Genetic testing was not routinely performed in this cohort; thus, patients were not classified as familial or sporadic. (2) Aged 18 years or older; (3) Newly diagnosed cases; (4) Having complete and accessible peripheral blood test data; (5) Not having received riluzole or edaravone treatment; (6) Not having used corticosteroids or anti-inflammatory drugs.

Healthy Control Group: (1) Healthy individuals with gender matching to the ALS patient group and assessed as having no neurological diseases; (2) Aged 18 years or older; (3) Having complete and accessible peripheral blood test data.

### Exclusion criteria

(1) Coexisting with other neurological diseases, such as Parkinson’s disease or multiple sclerosis; (2) Coexisting with severe metabolic diseases, such as diabetes or thyroid dysfunction; (3) Having used immunomodulatory drugs or glucocorticoids within the past 3 months; (4) Having severe infectious diseases, such as active tuberculosis or HIV infection; (5) Having severe liver or kidney dysfunction.

### Sample size calculation

Sample-size estimation was performed using Rosner’s method [18] and G*Power software. Based on a two-sample t-test comparing biomarker levels between groups, with an effect size (Cohen’s d) of 0.8, two-tailed α = 0.05, and power = 0.95, a minimum of 42 subjects per group was required. A total of 54 participants per group were ultimately enrolled, exceeding this requirement.

### Data collection

The collected data included basic demographic details such as age, gender of the participants, along with peripheral blood test results. Age is defined as the participant’s age at the time of blood sample collection. Blood Collection Procedure: Participants were instructed to fast overnight for 8 h. Between 6:00 and 7:00 AM, venous blood samples were collected from the elbow using sterile K2EDTA anticoagulant blood collection tubes (Hebei Xinle Medical Apparatus & Instruments Co., Ltd.). Subsequently, the blood samples were analyzed in the laboratory using an automated hematology analyzer (Unicel DxH800 Coulter Cellular Analysis System; Beckman Coulter, Inc.). The analyzed parameters included white blood cells, neutrophils, eosinophils, basophils, monocytes, lymphocytes, glycated haemoglobin A1c (HbA1c), fasting plasma glucose (FPG), and the haemoglobin glycation index (HGI). HGI is an indicator used to evaluate inter-individual differences in HbA1c levels, reflecting variations in the degree of hemoglobin glycation at a given blood glucose level. HGI is calculated by subtracting the predicted HbA1c value, derived from the average blood glucose (AG) level, from the laboratory-measured HbA1c value, using the formula: HGI = observed HbA1c − predicted HbA1c.

### Statistical analysis

Statistical analyses were performed using SPSS 25.0 and R 4.4.0. Continuous variables were tested for normality using the Shapiro-Wilk test. Variables following a normal distribution are reported as mean ± standard deviation (SD) and compared using the t-test; non-normally distributed variables are reported as median (M) with interquartile ranges (Q1, Q3) and compared using the Mann-Whitney U test. Categorical data are expressed as n (%) and evaluated with χ^2^ test. Spearman correlation analysis was used to evaluate the potential impact of age on biomarker levels. Logistic regression identified ALS-associated factors, and ROC curves assessed predictor performance. The AUC and its 95% confidence interval were calculated using the DeLong method. Random-forest and SHAP analyses supplemented these findings. The random forest model was built with 500 trees, and the number of variables randomly sampled at each split was set to the square root of the total number of predictors. Given the exploratory nature of this study and the testing of multiple biomarkers, we did not apply a correction for multiple comparisons to avoid an increased risk of type II errors and to prioritize the identification of potential signals for hypothesis generation. However, we acknowledge this as a methodological limitation, and the findings should be interpreted with caution. Significance was set at P < 0.05.

## Results

### Comparison of clinical data between the two groups

Table 1 compares the basic demographic information and peripheral blood test data of the participants in the two groups. The results revealed significant differences between the two groups in terms of age (*Z* = −3.232, *P* = 0.001), leukocyte (*t* = −2.165, *P* = 0.033), monocyte (*t* = 2.114, *P* = 0.037), HbA1c level (*t* = −2.873, *P* = 0.004), and HGI (*t* = −2.516, *P* = 0.014).

**TABLE 1:** Baseline data of the study participants.

Variables	Control group (n = 54)	ALS group (n = 54)	*t*/χ^2^/*Z*	*P*
Age [year, M (Q1, Q3)]	56.50 (46.00, 64.00)	63.00 (57.75, 68.25)	−3.232	0.001
Male [n (%)]	34 (62.96%)	36 (66.67%)	0.162	0.687
Hypertension [n (%)]	27 (50.00%)	27 (50.00%)	0.000	1.000
Leukocyte (×10^9^/L, mean ± SD)	5.86 ± 1.51	5.31 ± 1.14	−2.165	0.033
Neutrophil [×10^9^/L, M (Q1, Q3)]	3.37 (2.78, 4.00)	3.10 (2.41, 3.80)	−1.782	0.075
Eosinophil [×10^9^/L, M (Q1, Q3)]	0.12 (0.07, 0.18)	0.11 (0.07, 0.19)	−0.348	0.728
Basophil [×10^9^/L, M (Q1, Q3)]	0.03 (0.02, 0.05)	0.03 (0.02, 0.04)	−1.391	0.164
Monocyte (×10^9^/L, mean ± SD)	0.36 ± 0.07	0.39 ± 0.08	2.114	0.037
Lymphocyte (×10^9^/L, mean ± SD)	1.78 ± 0.55	1.68 ± 0.49	−1.028	0.306
HbA1c [%, M (Q1, Q3)]	5.80 (5.60, 6.10)	5.60 (5.38, 6.00)	−2.873	0.004
FPG [mmol/L, M (Q1, Q3)]	4.94 (4.72, 5.51)	5.05 (4.54, 5.35)	−0.578	0.563
HGI (mean ± SD)	−1.24 ± 0.34	−1.44 ± 0.47	−2.516	0.014

ALS, Amyotrophic lateral sclerosis; HbA1c, glycated haemoglobin A1c; FPG, fasting plasma glucose; HGI, haemoglobin glycation index. The same below.

### Spearman correlation analysis

To assess the potential influence of age on biomarker levels, Spearman correlation analysis was performed. As summarized in Table 2, age showed statistically significant positive correlations with monocyte count (*r* = 0.234, *P* = 0.015), HbA1c level (*r* = 0.327, *P* = 0.001), and HGI (*r* = 0.263, *P* = 0.006). In contrast, no significant correlation was observed between age and leukocyte count (*r* = −0.003, *P* = 0.972). These findings indicate that older age is associated with higher levels of monocytes, HbA1c, and HGI in the study population. However, leukocyte count appears to be independent of age in this cohort.

**TABLE 2:** Spearman correlation analysis.

Age	Leukocyte	Monocyte	HbA1c	HGI
*r*	−0.003	0.234	0.327	0.263
*P*	0.972	0.015	0.001	0.006

### Logistic regression exploration of ALS-related influences

**FIGURE 2:**
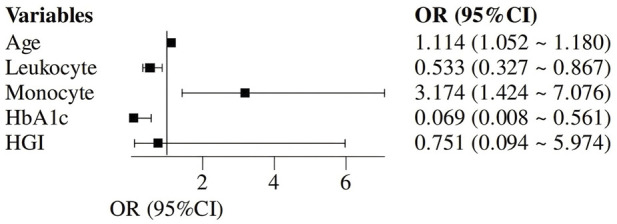
Distribution of OR Values and 1 in the Logistic regression analysis of factors influencing ALS.

Logistic regression analysis was conducted with age, leukocyte, monocyte, HbA1c, and HGI as independent variables, and ALS diagnosis as the dependent variable. The results demonstrated that age (OR = 1.114, 95% CI: 1.052–1.180, *P* < 0.001), leukocyte (OR = 0.533, 95% CI: 0.327–0.867, *P* = 0.011), monocyte (OR = 3.174, 95% CI: 1.424–7.076, *P* = 0.005), and HbA1c level (OR = 0.069, 95% CI: 0.008–0.561, *P* = 0.012) were significant factors influencing ALS. However, the logistic regression model included both HbA1c and HGI, which are mathematically related, potentially leading to multicollinearity and unstable estimates; the extremely low odds ratio for HbA1c with a wide confidence interval (0.008–0.561) suggests this finding should be interpreted with caution. See Table 3 and Figure 2.

**TABLE 3:** Logistic regression exploration of ALS-related influences.

Variables	β	SE	Wald	*P*	OR (95% CI)
Age	0.108	0.029	13.495	<0.001	1.114 (1.052–1.180)
Leukocyte	−0.630	0.249	6.412	0.011	0.533 (0.327–0.867)
Monocyte	1.155	0.409	7.974	0.005	3.174 (1.424–7.076)
HbA1c	−2.675	1.070	6.252	0.012	0.069 (0.008–0.561)
HGI	−0.287	1.058	0.073	0.786	0.751 (0.094–5.974)

OR, odds ratio; CI: Confidence Interval. The same below.

### Construction of a random forest model

**FIGURE 3:**
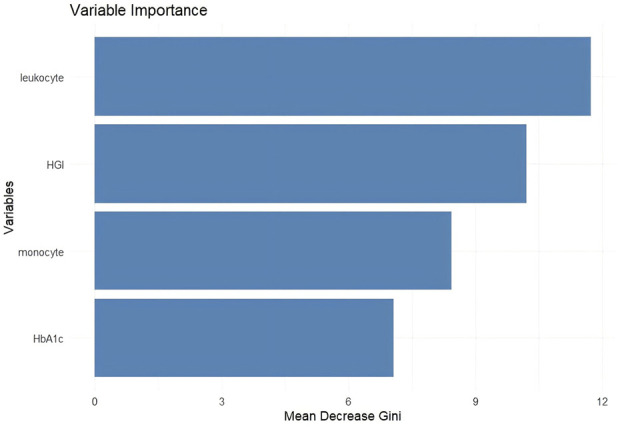
Significance ranking of factors influencing ALS in the random forest prediction model. Note: The random forest model was trained using peripheral blood and glycation biomarkers (leukocyte, monocyte, HbA1c, and HGI) as predictors. Variable importance was assessed using the mean decrease in accuracy (MDA), which quantifies the reduction in model accuracy when each variable is permuted. Higher MDA values indicate greater predictive importance. The x-axis shows the mean decrease in accuracy, and the y-axis lists the predictor variables in descending order of importance. Leukocyte count ranked highest, followed by HGI, monocyte, and HbA1c level.

**FIGURE 4:**
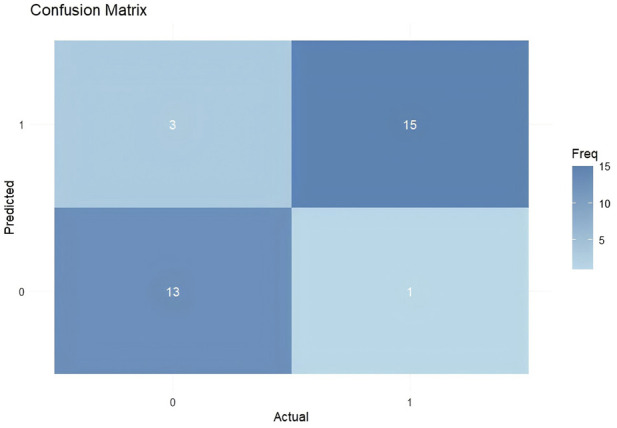
Confusion matrix of prediction accuracy in the random forest prediction model for ALS. Note: Predictions were generated using out-of-bag (OOB) samples to reduce overfitting. The matrix displays the number of correctly and incorrectly classified cases based on the random forest model. The x-axis represents the predicted classification (0: healthy control, 1: ALS), and the y-axis represents the actual classification (0: healthy control, 1: ALS). The color intensity corresponds to the frequency count, as indicated by the color bar.

The random forest algorithm was employed to rank the factors in Table 2 based on their controllability. The random forest model was trained using the full dataset, and predictive performance was evaluated using out-of-bag (OOB) predictions to minimize overfitting. Age was not included in the random forest model to focus the analysis on the diagnostic potential of the peripheral blood and glycation biomarkers. The results indicated that, in descending order of importance, leukocyte count, HGI, monocyte, and HbA1c level all had an impact on ALS (Figure 3). When using these indicators to predict ALS, the false-positive rate was 18%, and the false-negative rate was 6% (Figure 4).

### Construction of the ROC model

**FIGURE 5:**
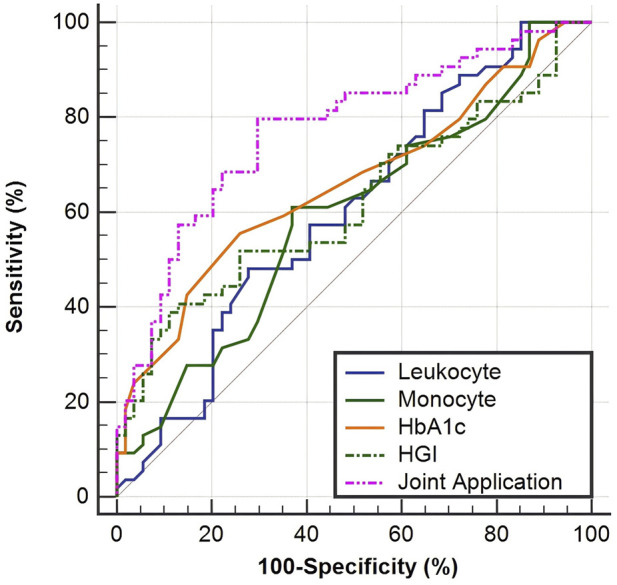
ROC curve of the prediction model for factors influencing ALS.

This study evaluated the predictive efficacy of four individual indicators—leukocytes, monocytes, HbA1c, and HGI—as well as their joint application for ALS through ROC curve analysis. As shown in Table 4 and Figure 5, among the individual indicators, HbA1c demonstrated an AUC of 0.660 (95% CI: 0.562–0.748), and HGI showed an AUC of 0.619 (95% CI: 0.521–0.711) (*P* < 0.05). The joint application of these indicators exhibited the highest diagnostic value, with an AUC of 0.774 (95% CI: 0.683–0.849), a sensitivity of 79.63%, and a specificity of 70.37%. Table 5 shows, via pairwise comparisons, that the joint application AUC significantly exceeded that of every single indicator (all *P* < 0.05), indicating that combining multiple indicators can significantly enhance the differential diagnostic capability for ALS.

**TABLE 4:** ROC curve analysis of the prediction model for factors influencing ALS.

Indicator	AUC	95% CI	Sensitivity	Specificity	Best cut-off value	*Z*	*P*
Leukocyte	0.602	0.504–0.695	48.15	72.22	≤5.15	1.865	0.062
Monocyte	0.592	0.493–0.686	61.11	62.96	>0.37	1.678	0.093
HbA1c	0.660	0.562–0.748	55.56	74.07	≤5.60	3.033	0.002
HGI	0.619	0.521–0.711	38.89	88.89	≤-1.58	2.168	0.030
Joint application	0.774	0.683–0.849	79.63	70.37	​	6.018	<0.001

The “joint application” refers to a combined logistic regression model incorporating leukocyte, monocyte, HbA1c, and HGI as predictors.

**TABLE 5:** Pairwise comparison of ROC curves.

Comparison	Difference between areas	95% CI	*Z*	*P*
Leukocyte∼joint application	0.171	0.054–0.289	2.864	0.004
Monocyte∼joint application	0.181	0.069–0.294	3.159	0.002
HbA1c∼joint application	0.114	0.019–0.209	2.345	0.019
HGI∼joint application	0.155	0.045–0.264	2.769	0.006

### Construction of the SHAP model

**FIGURE 6:**
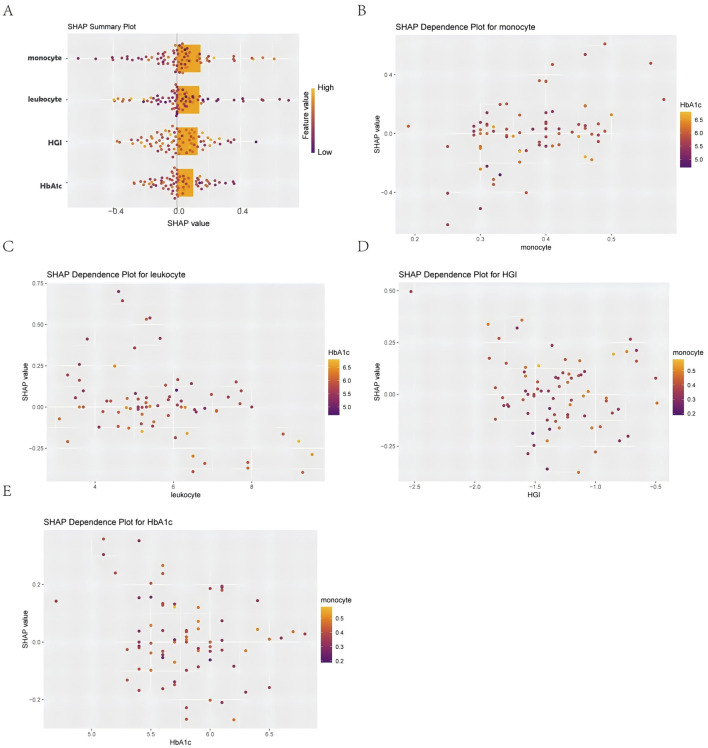
**(A)** SHAP summary plot for monocyte, leukocyte, HGI, and HbA1c. **(B)** SHAP dependence plot for monocyte. **(C)** SHAP dependence plot for leukocyte. **(D)** SHAP dependence plot for HGI. **(E)** SHAP dependence plot for HbA1c.

The SHAP analysis method was employed to visually interpret the selected variables and illustrate their contributions to the model’s identification results. Figure 6 displays the four feature variables included in the model: monocyte, leukocyte count, HGI, and HbA1c. A dot plot shows each variable’s contribution to the prediction, with yellow and purple dots indicating high- and low-risk values, respectively.

The analysis reveals that an elevation in monocyte, along with reductions in leukocyte count, HGI, and HbA1c levels, is associated with an increased risk of ALS onset. The bar chart ranks risk factors by their mean absolute SHAP values. The horizontal axis represents the SHAP values, where positive values enhance the probability of identification, whereas negative values decrease it. The vertical axis reflects the relative importance of each feature in the predictive model, with variables positioned higher being more important and those lower being less important. The results show that, in descending order of importance, the variables are: monocyte, leukocyte count, HGI, and HbA1c.

## Discussion

Evidence indicates that patients typically wait 12–18 months from first symptoms to a confirmed ALS diagnosis [19]. Diagnostic delays often result in patients missing the optimal window for early intervention, further exacerbating irreversible motor function impairment and significantly reducing both quality of life and survival duration. Therefore, identifying potential diagnostic biomarkers with high specificity and sensitivity, as well as establishing an efficient early diagnostic system, has emerged as one of the core research priorities in the field of ALS. This study focused on peripheral immune cells and glycosylation indices, systematically exploring their diagnostic value in ALS through multidimensional statistical analyses and model validations.

Initially, baseline data comparisons revealed significant statistical differences between the ALS and control group in terms of age, leukocyte count, monocyte, HbA1c level, and HGI. Regarding age, logistic regression analysis demonstrated that age was a risk factor for ALS (OR = 1.114, P < 0.001), indicating that for each additional year of age, the risk of developing ALS increased by approximately 11.4%. This finding aligns with the conclusions of most previous epidemiological studies, which have shown a clear upward trend in ALS incidence with advancing age, peaking particularly in the 50–70 age group [20–22]. The potential underlying reasons include the gradual decline of neuroprotective mechanisms, elevated oxidative stress levels, disrupted protein homeostasis, and reduced repair capacity of nerve cells with aging, all of which increase the vulnerability of neurons to damage and thereby raise the risk of neurodegenerative diseases [23]. These results also suggest that in clinical practice, when older people present with unexplained muscle weakness, atrophy, or other symptoms, a high index of suspicion for ALS is warranted, and of proceeding with targeted testing to confirm it.

This study found a significantly higher monocyte in the ALS group compared to healthy controls, and logistic regression revealed it as a risk factor for the disease. While previous research has implicated neuroinflammation in ALS pathogenesis [24], our findings are limited to demonstrating a statistical association between elevated peripheral monocyte counts and ALS. The biological mechanisms underlying this association remain to be elucidated and cannot be directly inferred from the current data. In contrast to monocytes, we observed that leukocytes were notably diminished in ALS patients relative to healthy individuals and were identified as a protective factor. We acknowledge that this finding differs from some previous studies, some of which have reported normal or elevated leukocyte counts in ALS patients [25, 26]. Rather than speculating on the reasons for these discrepancies, we emphasize that they may be attributable to differences in sample size, disease stage, clinical heterogeneity, or population characteristics across studies. In the present cohort, no significant differences were observed in neutrophil or lymphocyte counts between the two groups, suggesting that the NLR—a well-established prognostic marker in ALS—also did not differ. This observation further indicates that the lower total leukocyte count in our ALS group was not driven by meaningful shifts in these key immune subsets. Taken together, these findings highlight the need for future studies with larger, well-phenotyped cohorts and standardized protocols to clarify the role of peripheral immune cell profiles in ALS.

ALS is not only a neurodegenerative disease but also accompanied by significant systemic metabolic disturbances, among which glucose metabolic disorders represent a crucial manifestation. Both HbA1c levels and the HGI were significantly lower in the ALS group, but only HbA1c was identified as a protective factor for ALS via logistic regression. However, the random forest prediction model identified HGI as the second most important predictor of ALS, second only to leukocyte count. This discrepancy in assessing the importance of HGI may stem from differences in the underlying assumptions of the two models: unlike logistic regression, which assumes linearity, random forest captures nonlinear relationships and complex interactions among variables. We hypothesize that HbA1c and HGI may jointly participate in ALS pathogenesis through linear and nonlinear mechanisms, respectively. The association between decreased levels of HbA1c and HGI and an increased risk of ALS onset resonates with recent research on the metabolic mechanisms of ALS, suggesting that glucose metabolic disturbances may contribute to the pathological processes of ALS through multiple pathways.

A nationwide Danish study found a protective association between type 2 diabetes and ALS [27]. This was supported by a large case-control study, which found a significant inverse correlation specifically for non-insulin-dependent diabetes implying a potential ALS-protective effect [28]. Furthermore, multiple studies have confirmed that elevated blood glucose levels may delay the onset of ALS in diabetic patients [29–32]. Our findings corroborate those earlier observations, as HbA1c and HGI demonstrated significant predictive value in the models, indicating that decreased levels of these indicators might signal a higher chance of ALS occurrence. This could be because ALS patients often exhibit a hypermetabolic state, and hyperglycemia or diabetes may, to some extent, alleviate the neuronal energy crisis by providing more abundant energy substrates, thereby delaying disease onset [33]. Additionally, under hyperglycemic conditions, cells may activate the Nrf2 pathway, a key transcription factor regulating antioxidant stress responses. Activation of Nrf2 can reduce oxidative stress damage, which is an important mechanism underlying ALS pathogenesis [34]. It is important to note that not all forms of hyperglycemia are protective; for instance, the autoimmune form is instead linked to a higher ALS risk, suggesting that the presence of insulin may be a critical factor. Furthermore, although hyperglycemia may delay the onset of ALS, diabetic patients may experience faster disease progression and poorer prognosis after ALS diagnosis [35].

ROC curve analysis is a crucial method for evaluating the diagnostic performance of biomarkers. In this study, ROC curve analyses of both individual and combined indicators were conducted to clarify the diagnostic value of each indicator. Among the individual indicators, HbA1c demonstrated the highest diagnostic efficacy, followed by HGI, while the diagnostic efficacy of leukocyte count and monocyte was relatively low. Given that a single biomarker often struggles to comprehensively reflect the pathological processes of complex diseases, combining multiple indicators with distinct biological significances typically enhances diagnostic accuracy. The findings of this study corroborate this notion, as the combined diagnosis using four indicators—leukocytes, monocytes, HbA1c, and HGI—achieved an AUC of 0.774, with 79.63% sensitivity and 70.37% specificity. The combined detection integrates information on both immune inflammation and metabolic disturbances, providing a more comprehensive reflection of the pathological state of ALS and thereby improving diagnostic accuracy. The indicators involved in the combined detection are all peripheral blood tests, which offer advantages such as convenient sampling, minimal invasiveness, and low detection costs, facilitating their clinical application, particularly in primary hospitals and large-scale screening programs, and providing a feasible technical solution for the early diagnosis of ALS.

To comprehensively evaluate the impact of each candidate indicator on ALS diagnosis, this study also employed the random forest model and SHAP interpretability analysis, reinforcing the credibility of the outcomes. The random forest algorithm, through ensemble learning of multiple decision trees, effectively handles interactions among variables and assesses the importance of each indicator. The results showed that, in descending order of importance, leukocyte count, HGI, monocyte, and HbA1c level all influenced ALS diagnosis, which was largely consistent with the findings from logistic regression analysis and ROC curve analysis, further confirming the significant role of these indicators in ALS diagnosis. Meanwhile, the random forest model exhibited good predictive performance with low false-positive and false-negative rates, indicating its strong potential for clinical application as an auxiliary diagnostic tool for ALS. SHAP analysis, as an interpretability method based on game theory, gauges each variable’s effect on the model’s predictive performance and visually displays the relationship between each indicator and disease risk through charts. The SHAP analysis results in this study revealed that elevated monocyte and decreased leukocyte count, HGI, and HbA1c level were all associated with ALS onset, with the feature importance ranked in descending order as monocyte, leukocyte count, HGI, and HbA1c. This visual interpretation not only aids clinicians in better understanding the diagnostic significance of each indicator but also provides clear directions for subsequent mechanistic research.

For high-risk populations, such as those with a family history of ALS or unexplained muscle weakness, preliminary screening can be conducted through combined peripheral blood testing. If the model indicates a high risk, further examinations such as electromyography and cranial MRI can be performed, which can significantly reduce the misdiagnosis rate and shorten the diagnostic time. Additionally, dynamic monitoring of indicators such as monocytes and HbA1c during treatment can indirectly assess inflammatory and metabolic states. A decrease in monocyte may suggest effective anti-inflammatory treatment, while an increase in HbA1c may reflect improved energy metabolism, providing references for individualized treatment. Although this study has yielded valuable results, it still has some limitations. First, while logistic regression identified HbA1c as a potential protective factor, the extremely low odds ratio and wide confidence interval indicate considerable instability in this estimate. Moreover, HbA1c and the HGI are mathematically related, and their simultaneous inclusion in the same regression model may introduce multicollinearity, making it difficult to reliably disentangle their individual effects. Therefore, this finding should be interpreted with caution and considered exploratory, requiring validation in larger, independent cohorts. Second, the study’s relatively small sample size and single-center, retrospective design increase the risk of model overfitting. Although the random forest model utilized out-of-bag error for internal validation, the logistic regression model did not undergo any internal validation procedures, such as cross-validation, bootstrapping, or optimism correction. Consequently, the reliability and generalizability of our findings, particularly those from the logistic regression model, remain unproven and require external validation in larger, independent cohorts, such as the UK Biobank. Third, this study did not include patients with other neuromuscular diseases as controls. Therefore, we cannot conclude that the observed biomarker profile is specific to ALS. Future studies should incorporate disease control groups to evaluate the specificity of these biomarkers for distinguishing ALS from ALS-mimic conditions. Fourth, critical clinical parameters that are essential for a comprehensive understanding of ALS, such as site of onset, disease duration, genetic status, and progression rate, were not systematically collected in this retrospective cohort. Consequently, we were unable to explore the relationship between the candidate biomarkers and these key clinical characteristics, including their potential to reflect disease severity rather than merely serving as diagnostic indicators. This represents an important gap that should be addressed in future prospective studies. Lastly, this study did not explore the dynamic change patterns of the indicators. If their temporal sequences of change could be clarified, the value for early diagnosis could be further enhanced. Therefore, future research should conduct multi-center prospective cohort studies, expand the sample size, and include patients with underlying comorbidities to validate the stability and specificity of the combined model. Convenient diagnostic tools based on combined indicators can also be developed and clinically translated in primary hospitals to truly achieve early diagnosis and intervention for ALS. It is important to note that, given the above-mentioned limitations—particularly the retrospective design, single-center recruitment, lack of external validation, and absence of disease control groups—our findings should be considered exploratory. The proposed clinical application of the combined detection model requires further validation before it can be implemented in routine practice.

In summary, this study provides preliminary evidence that peripheral blood leukocytes, monocytes, HbA1c, and HGI may serve as potential diagnostic biomarkers for ALS. Combined detection of these indicators demonstrated improved diagnostic accuracy in this cohort. However, given the exploratory nature of the findings and the limitations of the study design, these results should be interpreted with caution. Further validation in larger, independent cohorts—including disease control groups—is required to confirm the specificity and robustness of these biomarkers before they can be considered for clinical application.

## Data Availability

The original contributions presented in the study are included in the article/supplementary material, further inquiries can be directed to the corresponding authors.
